# Linking Multi-Layer Dynamical GCN With Style-Based Recalibration CNN for EEG-Based Emotion Recognition

**DOI:** 10.3389/fnbot.2022.834952

**Published:** 2022-02-24

**Authors:** Guangcheng Bao, Kai Yang, Li Tong, Jun Shu, Rongkai Zhang, Linyuan Wang, Bin Yan, Ying Zeng

**Affiliations:** ^1^Henan Key Laboratory of Imaging and Intelligent Processing, PLA Strategic Support Force Information Engineering University, Zhengzhou, China; ^2^Key Laboratory for NeuroInformation of Ministry of Education, School of Life Science and Technology, University of Electronic Science and Technology of China, Chengdu, China

**Keywords:** electroencephalography (EEG), emotion recognition, graph convolutional neural networks (GCNN), convolutional neural networks (CNN), style-based recalibration module (SRM)

## Abstract

Electroencephalography (EEG)-based emotion computing has become one of the research hotspots of human-computer interaction (HCI). However, it is difficult to effectively learn the interactions between brain regions in emotional states by using traditional convolutional neural networks because there is information transmission between neurons, which constitutes the brain network structure. In this paper, we proposed a novel model combining graph convolutional network and convolutional neural network, namely MDGCN-SRCNN, aiming to fully extract features of channel connectivity in different receptive fields and deep layer abstract features to distinguish different emotions. Particularly, we add style-based recalibration module to CNN to extract deep layer features, which can better select features that are highly related to emotion. We conducted two individual experiments on SEED data set and SEED-IV data set, respectively, and the experiments proved the effectiveness of MDGCN-SRCNN model. The recognition accuracy on SEED and SEED-IV is 95.08 and 85.52%, respectively. Our model has better performance than other state-of-art methods. In addition, by visualizing the distribution of different layers features, we prove that the combination of shallow layer and deep layer features can effectively improve the recognition performance. Finally, we verified the important brain regions and the connection relationships between channels for emotion generation by analyzing the connection weights between channels after model learning.

## Introduction

Human emotion is a state that reflects the complex mental activities of human beings. In recent years, new modes of human-computer interaction, such as voice, gesture, and force feedback, have sprung up. Although significant progress has been made in the field of human-computer interaction, it still lacks one of the indispensable functions of human-computer interaction, emotional interaction (Sebe et al., 2005). However, the prerequisite for realizing human-computer emotional interaction is to recognize human emotional state in real time. Human emotions come in many forms, which can be recognized by human facial expressions (Harit et al., 2018), body movements (Ajili et al., 2019), and physiological signals (Goshvarpour and Goshvarpour, 2019; Valderas et al., 2019). But humans can control their facial expressions, body movements to hide or disguise their emotions, and physiological signals such as electroencephalogram, electrocardiogram, and electromyography have the advantage of being difficult to hide or disguise. With the rapid development of non-invasive, portable, and inexpensive EEG acquisition equipment, EEG-based emotion recognition has attracted the attention of researchers.

EEG signals are collected through electrodes distributed in various brain regions on the cerebral cortex, which has the advantages of non-invasiveness, convenience, and fast. In addition, EEG have the advantages of high time resolution, and are considered to be one of the most reliable signals. However, EEG also has some shortcomings, such as low spatial resolution and low signal-to-noise ratio. Moreover, the EEG is non-stationary, and there are great differences among subjects. Studies have shown that some cortical and subcortical brain systems may play a key role in the evaluation or reaction phase of emotion generation (Clore and Ortony, 2008; Kober et al., 2008). However, it is difficult to use EEG to model brain activity and interpret the activity state of brain regions. Therefore, high-precision recognition of emotions based on EEG is still a challenge.

In these decades of development, researchers have proposed many machine learning and signal processing methods for EEG emotion recognition. Traditional EEG emotion recognition methods usually include two aspects: EEG feature extraction and emotion classification used to distinguish emotion categories. The EEG features used for emotion recognition are mainly divided into three parts: time-domain features, frequency-domain features, and time-frequency features. Time domain features mainly include statistics (Jenke et al., 2017), Hjorth features (Hjorth, 1970), non-stationary index (NSI) (Kroupi et al., 2011), fractal dimension (Sourina and Liu, 2011; Liu and Sourina, 2013), sample entropy (Jie et al., 2014), and higher order crossings (HOC) (Petrantonakis and Hadjileontiadis, 2011). These features mainly describe the temporal characteristics and complexity of EEG signals. Frequency domain feature refers to the use of Fourier Transform (TF) and other information analysis methods to transform EEG signals from time domain to frequency domain, and then extract emotion related information from frequency domain as features. At present, one of the most commonly used frequency domain feature extraction methods is to divide EEG signals into five bands: Delta (1–4 Hz), Theta (4–8 Hz), Alpha (8–12 Hz), Beta (12–30 Hz), Gamma (30–64 Hz). Emotion Feature Extraction in frequency domain mainly includes power spectral density (PSD) (Alsolamy and Fattouh, 2016), differential entropy (DE) (Duan et al., 2013), differential asymmetry (DASM) (Liu and Sourina, 2013), rational asymmetry (RASM) (Lin et al., 2010), and differential causality (DCAU) (Zheng and Lu, 2015). Time frequency feature refers to the use of time-frequency analysis methods, such as short-time Fourier transform (STFT) (Lin et al., 2010), wavelet transform (WT) (Jatupaiboon et al., 2013) and Hilbert Huang transform (HHT) (Hadjidimitriou and Hadjileontiadis, 2012). Due to the typical non-stationary signal of EEG, the traditional frequency domain analysis method such as Fourier transform is not suitable for analyzing the signal whose frequency changes with time, while the time-frequency analysis method provides the joint distribution information of time domain and frequency domain.

The classifiers based on EEG emotion recognition are mainly divided into traditional machine learning method and deep network method. Among the traditional machine learning methods, support vector machine (SVM) (Koelstra et al., 2010; Hatamikia et al., 2014), k-nearest neighbor (KNN) (Mehmood and Lee, 2015), linear discriminant analysis (LDA) (Zong et al., 2016) and other methods are used for emotion classification based on EEG. Among them, SVM has better performance and is usually used as baseline classifier. However, due to the complexity of EEG-based emotion features, the current method is to extract the artificial features, and then use machine learning method to classify the extracted features, which leads to the traditional machine learning method cannot get better classification performance. Therefore, researchers turn their attention to deep learning methods. Zhang X. et al. (2019) summarized the work of using deep learning technology to study brain signals in recent years. In EEG-based emotion recognition based on neural network, the input is usually artificial features, and then the neural network is used to learn deeper features to improve the performance of emotion recognition. Zheng et al. (2014) used deep belief networks (DBNs) to learn and classify the frequency bands and channels of EEG-based emotion, which is a great improvement compared to SVM. In recent years, many deep networks have emerged in this field to extract spatiotemporal features of EEG-based emotions. Jia et al. (2020) proposed a spatial-spectral-temporal based Attention 3D Dense Network (SST-EmotionNet) for EEG emotion recognition. Li Y. et al. (2018) and Li et al. (2020) proposed BiDANN and BiHDM networks for EEG emotion recognition, considering the asymmetry of emotion response between left and right hemispheres of human brain. Li et al. (2021) proposed a Transferable Attention Neural Network (TANN), which considers local and global attention mechanism information for emotion recognition. In addition, some researchers considered the spatial information of EEG features, and arrange and distribute the features of each channel through the physical location before inputting them into the neural network. Li J. et al. (2018) arranged the DE features of different leads into a two-dimensisonal feature matrix according to their physical locations before entering the network. Bao et al. (2021) mapped the DE feature to a two-dimensional feature matrix through an interpolation algorithm according to the physical location.

Although researchers currently use neural network to consider the temporal and spatial information, the EEG signals of each channel are distributed in different regions of the brain, which can be regarded as a non-Euclidean data. However, convolution neural network processing EEG will ignore the spatial distribution information. In order to solve this problem, graph convolution neural network (GCNN) (Defferrard et al., 2016) is introduced to process non-Euclidean data. Zhao et al. (2022) proposed a new dynamic graph convolutional network (dGCN) to learn the potentially important topological information. Song et al. (2020) used dynamic graph convolution network (DGCNN) for the first time in the EEG-based emotion recognition task. The network constructed graph data more in line with the brain activity state by learning the connections between different channels, and achieved better performance. Zhong et al. (2020) proposed a regularized graph neural network (RGNN), which considers the global and local relationships of different EEG channels. Zhang T. et al. (2019) proposed GCB-Net, which combines GCN and CNN to extract deep-level features and introduces a generalized learning system (BLS) to further improve performance.

However, the brain activity in emotional state is more complex, and multiple brain regions participate in interaction. The traditional convolutional neural network cannot effectively learn the interaction between brain regions.

However, the networks proposed in the above studies all use one layer of GCN, and (Kipf and Welling, 2017) concluded that using 2-3 layers is the best. In addition, the receptive field of single-layer GCN is limited and cannot extract spatial information well. The brain activity in emotional state is more complex, and multiple brain regions participate in interaction. Therefore, the characteristics of single network learning are relatively single, and cannot well reflect the complex emotional state. For this reason, in this paper, we proposed a multi-layer dynamic graph convolutional network-style-based recalibration convolutional neural network (MDGCN-SRCNN) to extract shallow layer and deep layer features. The shallow layer features include the features of different levels of GCN learning, which contain different levels of spatial information. Deep layer features are mainly learned by SRCNN, because CNN has a strong ability to learn abstract features. In addition, by adding the style-based recalibration module, when CNN extracts features, it emphasizes the information related to emotion and ignores other information, which greatly enhances the representation ability of CNN. The shallow layer and deep layer features are connected to form a multi-level rich feature, and finally the fully connected layer search is used to classify the features that are distinguishable from various emotions.

The main contributions of this paper are as follows:

1. MDGCN-SRCNN framework composed of multi-layer GCN and multi-layer style-based recalibration CNN is used to learn features at different levels. In the shallow layer network, GCN learns different levels of spatial features. In the deep layer network, CNN learns abstract features, using a fully connected layer to fuse the shallow layer spatial features with deep layer abstract features and search for highly distinguishable features for emotion classification.
2. SEED and SEED-IV data sets are used to verify the performance of the emotion recognition framework MDGCN-SRCNN proposed in this paper. Compared with the existing models, the framework proposed in this paper obtains the best results, which proves that the network proposed in this paper has a strong classification ability in EEG emotion recognition.

## Methods

In this section, we introduce in detail the framework MDGCN-SRCNN proposed in this paper.

### Model Framework

**Figure 1:**
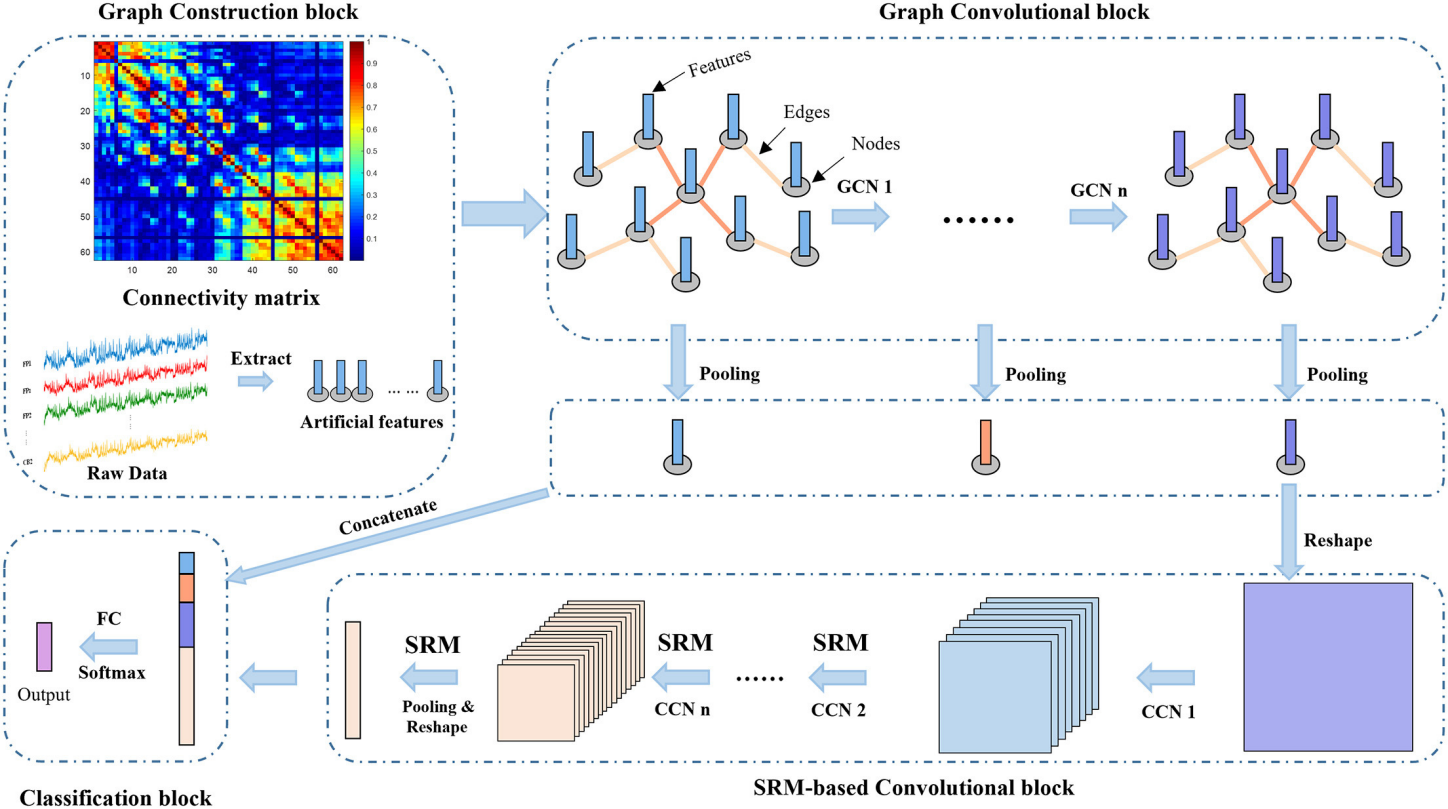
The overall architecture of the MDGCN-SRCNN model consists of four blocks: graph construction block, graph convolutional block, SRM-based convolutional block, and classification block. The output of the model is a predicted label with probability.

As shown in Figure 1, we propose the MDGCN-SRCNN framework for EEG-based emotion recognition tasks. The MDGCN-SRCNN model consists of four blocks: graph construction block, graph convolutional block, SRM-based convolutional block and classification block. We will give the specific model architecture below.

#### Graph Construction Block

We considered that EEG is non-Euclidean data. EEG data is collected by many electrodes, which are distributed in different parts of the brain. The construction of a graph requires three parts: nodes, features, and edge sets. For EEG signals, the nodes of the graph are the EEG signal channels. Different acquisition devices have different channel numbers. Currently, 16 channels, 32 channels, 64 channels, and 128 channels are commonly used.

The feature is the data collected by each channel, which can be the original collected data or manually extracted features. Most of the current researches use artificial features for EEG-based emotion recognition. Therefore, in this paper, the DE features of five bands are extracted as the features of the graph. Short Time Fourier Transform (STFT) is used to transform each segment of data. The formula of DE features is as follows:


$$\begin{array}{l} h\left(X\right)=-\int_{\infty }^{\infty }\frac{1}{\sqrt{2\pi \sigma^{2}}}e^{-\frac{{\left(x-\mu \right)}^{2}}{2\sigma^{2}}}\log\left(\frac{1}{\sqrt{2\pi \sigma^{2}}}e^{-\frac{{\left(x-\mu \right)}^{2}}{2\sigma^{2}}}\right)dx \end{array}$$

(1) $$\begin{array}{r} =\frac{1}{2}\log\left(2\pi e\sigma^{2}\right) \end{array}$$

where *X* ~ *N*(μ, σ^2^) is the input raw signal, *x* is a variable, and *e* and π are constants.

The edge set of the graph describes the connected relationship between nodes. Currently, Pearson correlation coefficient (PCC) (Faskowitz et al., 2020), coherence value (Wagh and Varatharajah, 2020), phase locked value (PLV) (Wang et al., 2019), and physical distance (Song et al., 2020) are mainly used to describe the connection between channels. In this paper, PCC is used as the weighted adjacency matrix of each channel, and its calculation formula is as follows:


(2) $$\begin{array}{r} A\left(i,j\right)=abs\left(PCC\left(x_{i},x_{j}\right)\right)=abs\left(\frac{cov\left(x_{i},x_{j}\right)}{\sigma_{x_{i}}\sigma_{x_{j}}}\right) \end{array}$$

where *i, j* = 1, 2, ......, *n*, *n* are the number of channels of EEG signals. *x*_*i*/*j*_ represents the EEG signal of the *i*/*j*-th channel. *cov*(·) refers to covariance.

#### Graph Convolutional Block

In the graph convolutional block, we use graph convolution network as a shallow layer network to learn the spatial information of EEG signals.

The graph convolutional neural network is the network using convolution operations on the graph. Given a graph G=(V,E), where V refers to the vertex set with |V|=n nodes, and E is a set of edges between nodes. Data on vertex V can be represented by a set of feature matrix *X* ∈ ℝ^*n*×*f*^, where *n* represents the number of nodes and *f* represents the feature dimension. The edge set E can be represented by a set of weighted adjacency matrices *A* ∈ ℝ^*n*×*n*^ describing the connections between nodes. Kipf and Welling (2017) proposed the propagation rules of Graph Convolutional Networks (GCN):


(3) $$\begin{array}{r} {\text{H}}^{\left(l+1\right)}=\sigma \left({\tilde{\text{D}}}^{-\frac{1}{2}}\tilde{\text{A}}{\tilde{\text{D}}}^{-\frac{1}{2}}{\text{H}}^{\left(l\right)}{\text{W}}^{\left(l\right)}\right) \end{array}$$

where $\tilde{\text{A}}=\text{A}+\text{I}$ is the adjacency matrix of the undirected graph G with additional self-connections, and **I** is the identity matrix. $\tilde{\text{D}}$ is the diagonal matrix of $\tilde{\text{A}}$, that is, ${\tilde{\text{D}}}_{\text{i}\text{i}}=\sum_{j}{\tilde{\text{A}}}_{\text{i}\text{j}}$, **W**^(*l*)^ is the training parameter matrix of the *l*-th layer. **H**^(*l*)^ is the transformation matrix of the *l*-th layer. σ refers to the activation function.

Next, GCN is analyzed by spectral convolution. The Laplacian operator matrix of the graph G is defined as **L** = **D**−**A**, the normalized Laplacian operator can be expressed as $\hat{\text{L}}=\text{I}-{\text{D}}^{-\frac{1}{2}}\text{A}{\text{D}}^{-\frac{1}{2}}$, and the characteristic decomposition of $\hat{\text{L}}$ is $\hat{\text{L}}=\text{U}\lambda {\text{U}}^{\tau }$, where **U** is the orthonormal eigenvector matrix, and **Λ** = *diag*(λ_1_, ..., λ_*n*_) is the diagonal matrix of the corresponding characteristic.

For the input signal *X*, the graph Fourier Transform is:


(4) $$\begin{array}{r} \hat{X}={\text{U}}^{T}X \end{array}$$

The inverse Fourier transform is as follows:


(5) $$\begin{array}{r} X=\text{U}\hat{X} \end{array}$$

The generalized convolution on the graph can be defined as the product of signal X and filter *g*_θ_ in Fourier domain:


(6) $$\begin{array}{r} g_{\theta }*\text{X}=\text{U}\left(\left({\text{U}}^{T}g_{\theta }\right)⊙\left({\text{U}}^{T}\text{X}\right)\right)=\text{U}g_{\theta }\left(\Lambda \right){\text{U}}^{T}\text{X} \end{array}$$

where ⊙ refers to the element-wise multiplication, and *g*_θ_(**Λ**) = *diag*(_*g*_θ__1__, ...,_*g*_θ_*n*_)represents the diagonal matrix with*n*spectral filtering coefficients.

If formula 6 is calculated directly, the amount of calculation is very large. For a large graph, it costs a lot to calculate all the features of Laplacian matrix, and it needs$O\left(n^{2}\right)$times to multiply with Fourier basis**U**. Therefore, Defferrard et al. (2016) proposed that the diagonal matrix *g*_θ_(**Λ**) of spectral filtering coefficients can be approximated to *K*^*th*^ by the truncated expansion of Chebyshev polynomials:


(7) $$\begin{array}{r} g_{\theta }\left(\Lambda \right)\approx \overset{K}{\underset{k=0}{\sum }}\theta_{k}T_{k}\left(\tilde{\Lambda }\right) \end{array}$$

where, $\tilde{\Lambda }=\frac{2}{\lambda_{\max}}\Lambda -\text{I}$, λ_max_ refer to the largest eigenvalues of L. θ is a vector of Chebyshev coefficients. Chebyshev polynomials *T*_*k*_(·) can be recursively computed as *T*_*k*_(*x*) = 2*xT*_*k*−1_(*x*) − *T*_*k*−2_(*x*), where *T*_0_(*x*) = 1 and *T*_1_(*x*) = *x*. Then the graph filtering operation can be written as:


(8) $$\begin{array}{r} g_{\theta }*\text{X}\approx \overset{K}{\underset{k=0}{\sum }}\theta_{k}T_{k}\left(\tilde{\text{L}}\right)\text{X} \end{array}$$

where $\tilde{\text{L}}=\frac{2}{\lambda_{\max}}\tilde{\text{L}}-\text{I}$ is the normalized Laplacian. Then equation 8 is the Laplacian polynomial. In this case, the computational complexity is reduced to O(|E|).

The GCN proposed by Thomas et al., based on Equation 8, sets *K* = 1, λ_max_ = 2, θ_0_ = −θ_1_, then Equation 8 becomes Equation 3.

The EEG signal is converted into graph structure data by graph construction block and input into graph convolution network. Assuming that the initial data of the input graph rolled into the network is **H**^(0)^, the output of the *l*-th graph convolutional layer is shown in formula 3.

#### SRM-Based Convolutional Block

In the SRM-based convolutional block, we use a convolutional neural network combined with a style-based recalibration module as a deep layer network to learn abstract features related to emotions. The style-based recalibration module can be regarded as an attention module. But different from the traditional attention mechanism, the style-based recalibration module dynamically learns the recalibration weight of each channel based on the importance of the task style, and then merges these styles into the feature map, which can effectively enhance the representation ability of convolutional neural network.

**Figure 2:**
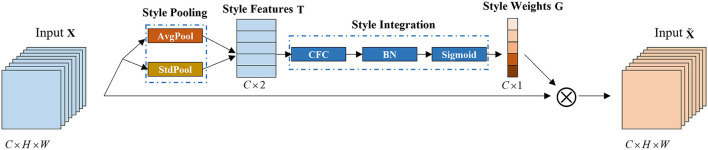
SRM module. This module is mainly composed of two parts: style pooling and style integration. AvgPool refers to global average pooling, StdPool refers to global standard deviation pooling; CFC refers to the channel fully connected layer; BN refers to batch standardization.

Given an input *X* ∈ ℝ^*N*×*C*×*H*×*W*^, SMR generates a channel-based recalibration weight *G* ∈ ℝ^*N*×*C*^ through the style of X, where*N*refers to the number of samples in the minimum batch training, *C* represents the number of channels, *H* and *W* represent the spatial dimensions. This module is mainly composed of style pooling and style integration, as shown in Figure 2.

In the style pooling module, using the mean and standard deviation of the channel as style features, the extracted style features are *T* ∈ ℝ^*N*×*C*×2^. Compared with other types of style features, using the mean and standard deviation of the channel can better describe the overall style information of each sample and channel (Lee et al., 2019). In the style integration module, the style features are converted into channel-related style weights through the channel fully connected layer, batch standard layer, and sigmoid activation function, which can simulate the importance of styles related to a single channel, thereby emphasizing, or suppressing them accordingly.

The output **H**^(*l*)^ of the convolutional network from the *l*-th graph is globally superposed and pooled as the input of the convolutional neural network, and then SRM is used in the middle of the convolutional layer to extract information related to the task style. Then each convolutional layer can be written as:


(9) $$\begin{array}{r} C_{k}=SRM\left(conv\left(C_{k-1},h\right)\right) \end{array}$$

where *h* = 1, 2, 3 represents the size of convolution kernel dimension, which is related to the input data type. In this paper, *h* = 2, $C_{0}=Pool\left({\text{H}}^{\left(l\right)}\right)$. *conv*(·, *h*) refers to *h*-dimensional convolution operation. *k* is the number of convolution layers. *SRM*(·) refers to SRM operation.

#### Classification Block

In the classification block, the learned features are input to the multi-layer fully connected layer for feature aggregation, and then the softmax layer is used for classification. After the shallow layer features and deep layer features are extracted, the multi-level features are spliced together, then the connected features can be written as:


(10) $$\begin{array}{r} \text{F}=\left[Pool\left({\text{H}}^{\left(1\right)}\right),Pool\left({\text{H}}^{\left(2\right)}\right),...,Pool\left({\text{H}}^{\left(l\right)}\right),Pool\left(C_{k}\right)\right] \end{array}$$

where *Pool*(·) refers to the global pooling operation, in which the global sum pooling operation is used in the graph convolutional network. Compared with the maximum pooling and the average pooling, the sum pooling shows a stronger expressive ability (Xu et al., 2019). In convolutional neural networks, maximum pooling is used.

The classification prediction of the input EEG signal is:


(11) $$\begin{array}{r} ŷ=softmax\left(FC\left(\text{F}\right)\right) \end{array}$$

where *FC*(·) refers to the fully connected layer operation, and ŷ ∈ ℝ^*C*^ is the predicted label of class *C*.

We use DGCNN to learn the adjacency matrix of the graph by optimizing the loss function. Then use the optimizer to optimize the cross entropy loss:


(12) $$\begin{array}{r} L=cross\text{\_}entropy\left(y,ŷ\right)+\alpha ||\Theta |{|}_{2} \end{array}$$

where *y* refers to the true label of the sample. θ is the matrix of all the parameters learned in the MDGCN-SRCNN model, α is the regularization coefficient, *cross*_*entropy*(·) refers to the calculation of cross entropy, and ||·||_2_ refers to the calculation of the second norm.

We use the Adam optimizer to learn the adjacency matrix**A**:


(13) $$\begin{array}{r} {\text{A}}^{*}=\text{A}-lr\frac{{\hat{m}}^{*}}{\sqrt{{\hat{v}}^{*}}+\varepsilon } \end{array}$$

(14) $$\begin{array}{r} {\hat{m}}^{*}=\frac{m^{*}}{1-\beta_{1}}=\frac{\beta_{1}m+\left(1-\beta_{1}\right)\nabla_{\theta }\theta }{1-\beta_{1}} \end{array}$$

(15) $$\begin{array}{r} {\hat{v}}^{*}=\frac{v^{*}}{1-\beta_{2}}=\frac{\beta_{2}v+\left(1-\beta_{2}\right){\left(\nabla_{\theta }\theta \right)}^{2}}{1-\beta_{2}} \end{array}$$

where A^*^ is the adjacency matrix after learning and **A** is initialization value. *lr* is learning rate. *m* = 0, *v* = 0, β_1_ = 0.9, β_2_ = 0.999, ε = 10^−8^. **θ** is all parameters of the network.

Algorithm 1 summarizes the specific implementation steps of the MDGCN-SRCNN model.

**Algorithm 1:** The training process of MDGCN-SRCNN.

**Input** : A labeled training data set$\left\{\text{X},\text{Y}\right\}={\left\{x_{i},y_{i}\right\}}_{i=1}^{N}$, the maximum number of training epochs T; the initialize adjacency matrix **A**,regularization coefficientα.
**Output**: The learned adjacency matrix$\hat{\text{A}}$, the model parameterΘ for MDGCN-SRCNN and the predicted labelŷ.
**Step 1** : Initialize the model parametersΘ in MDGCN-SRCNN model. Set iteration unit iter = 1;
**Step 2** : **while**iter < T **do**
**Step 3** : **for***k* = 1, ..., *l* **do**
**Step 4** : Calculate the *k*-th graph convolutional layer **H**^(*k*)^via Eq. (1) and calculate the *k*-th sum pooling layer*Pool*(**H**^(*k*)^);
**Step 5** : **for***k* = 1, ..., *l* **do**
**Step 6** : Calculate the *k*-th SMR-based convolution layer*C*_*k*_via Eq. (9);
**Step 7** : Concatenate the different layers of features **F**via Eq. (10);
**Step 8** : Calculate the prediction labelŷ via Eq. (11);
**Step 9** : Update the adjacency matrix **A**and the model parametersΘ via optimizer according to the cross-entropy loss.
**Step 10**: iter =iter+1;
**Step 11**: **end while**

### Details of the MDGCN-SRCNN Model

We consider that the amount of EEG data is too small, so the network cannot be designed too deep to prevent overfitting. In addition, the graph convolutional network cannot be superimposed too much, which will affect the performance, generally within 5 layers. After a small amount of trial and error experiments, we have observed that MDGCN-SRCNN achieves a higher accuracy rate under the two-layer graph convolutional layer and the two-layer convolutional layer plus the three-layer fully connected layer. The detailed description of the MDGCN-SRCNN model is shown in Table 1.

**Table 1:** MDGCN-SRCNN architecture.

**Block**	**Layer**	**Kernel size**	**Stride**	**Input**	**Output**	**Activation**
Graph convolution	Input				(*n, f*)	
	GCN1			(*n, f*)	(*n*, 16)	Leaky_ReLU
	Global_add_pool			(*n*, 16)	16	
	GCN2			(*n*, 16)	(*n*, 64)	Leaky_ReLU
	Global_add_pool			(*n*, 64)	64	
SMR-based convolution	Reshape			64	(8,8,1)	
	Conv1	(2,2)	2	(8,8,1)	(7,7,16)	Leaky_ReLU
	SMR1			(7,7,16)	(7,7,16)	Sigmoid
	Conv2	(2,2)	2	(7,7,16)	(6,6,32)	Leaky_ReLU
	SMR1			(6,6,32)	(6,6,32)	Sigmoid
	Max_pool	(2,2)		(6,6,32)	(3,3,32)	
Classifier	Reshape			(3,3,32)	3*3*32	
	FC1			16+64+3*3*32	256	Leaky_ReLU
	FC2			256	128	Leaky_ReLU
	FC3			128	C	Softmax

## Experimental Settings

In this section, we introduce the data sets and model settings used in the experiment.

### Datasets

We used two datasets SEED (Zheng and Lu, 2015) and SEED-IV (Zheng et al., 2018) to evaluate our proposed model.

#### SEED

The SEED data set contains EEG data of 15 subjects (7 males and 8 females), which were collected through 62 channels of ESI neuroscan system when they watched movie clips. All participants watched 15 movie clips, which contained five positive emotions, five neutral emotions, and five negative emotions. Each movie clip lasted about 4 min. There were three periods of data collection, and each subject collected a total of 45 experiments. The original EEG data were de sampled and the artifacts such as EOG and EMG were removed. The EEG data of each channel is divided into 1s segments without overlapping, and then the differential entropy characteristics of the five bands (Delta, Theta, Alpha, Beta, and Gamma) of the linear dynamic system smoothing (LDS) (Duan et al., 2013) are calculated for the segmented data segments.

#### SEED-IV

The EEG data of 15 healthy subjects (7 males and 8 females) were collected in the SEED-IV dataset using the same equipment as the SEED dataset. The data set selected 72 video clips to induce four different emotions (happy, neutral, sad, and fear). Each video clip lasted about 2 min. Each experiment conducted 24 experiments (6 experiments for each emotion). Each subject participated in three experiments at different times, and a total of 72 experiments were collected. Each experiment was divided into non-overlapping data segments of 4 s, each segment of data as a sample. Same as SEED, the differential entropy characteristics of five frequency bands are calculated.

### Model Settings

The parameter selection of the MDGCN-SRCNN model is based on previous experience and a small number of experiments. The Adam optimizer is used to optimize the loss function, and the learning rate is selected in the range of [0.001, 0.01]. L2 regular term coefficient α = 0.01. The fully connected layer in the SMR-based convolution block uses a dropout rate of 0.7. In the SEED data set, the batch size used is 16, and in SEED-IV, the batch size used is 9.

## Results and Analysis

In this section, we will evaluate the effectiveness and advancement of the propos ed model on the two data sets described in section Experimental Settings.

### Overall Performance

#### Performance on SEED

In the SEED data set, we refer to the settings of Zheng and Lu (2015), Song et al. (2020), and Li Y. et al. (2018). Each subject contains 15 trials per experiment. Therefore, the first 9 trials are used as the training set and the remaining 6 trials are used as the test set. The final accuracy and variance are the average results of 15 subjects.

The MDGCN-SRCNN model proposed in this paper is compared with the latest methods such as Support Vector Machine (SVM), Deep Belief Network (DBN), DGCNN, RGNN, GCB-net, STRNN, and BiHDM. In addition, we evaluated the performance of the related model on the 5 frequency bands of the DE feature. The comparison results of these models are shown in Table 2.

**Table 2:** Compare the accuracy rate (mean/std) with different existing methods on the SEED data set.

**Model**	**Delta band**	**Theta band**	**Alpha band**	**Beta band**	**Gamma band**	**All bands**
SVM (Zheng and Lu, 2015)	60.50/14.14	60.95/10.20	66.64/14.41	80.76/115.6	79.56/11.38	83.99/9.72
GSCCA (Zheng, 2017)	63.92/11.16	64.64/10.33	70.10/14.76	76.93/11.00	77.98/10.72	82.96/9.95
DBN (Zheng and Lu, 2015)	64.32/12.45	60.77/10.42	64.01/15.97	78.92/12.48	79.19/14.58	86.08/8.34
STRNN (Zhang et al., 2017)	**80.90/12.27**	**83.35/9.15**	**82.69/12.99**	83.41/10.16	69.61/15.65	89.50/7.63
GCNN (Song et al., 2020)	72.75/10.85	74.40/8.23	73.46/12.17	83.24/9.93	83.36/9.43	87.40/9.20
DGCNN (Song et al., 2020)	74.25/11.42	71.52/5.99	74.43/12.16	83.65/10.17	85.73/10.64	90.40/8.49
BiDANN (Li Y. et al., 2018)	76.97/10.95	75.56/7.88	81.03/11.74	**89.65/9.59**	88.64/9.46	92.38/7.04
GCB-net (Zhang T. et al., 2019)	80.38/10.04	76.09/7.54	81.36/11.44	88.05/9.84	88.45/9.67	92.30/7.40
GCB-net+BLS (Zhang T. et al., 2019)	79.98/8.93	76.51/9.56	81.97/11.05	89.06/8.69	89.10/9.55	94.24/6.70
RGNN (Zhong et al., 2020)	76.17/7.91	72.26/7.25	75.33/8.85	84.25/12.54	**89.23/8.9**	94.24/**5.95**
MDGCN-SRCNN	77.73/10.23	77.27/9.38	80.47/13.22	87.59/12.13	89.02/9.13	**95.08/**6.12

*Bold represents the best result*.

It can be seen in Table 2 that the model MDGCN-SRCNN proposed in this paper has achieved the best performance in the full-band features, with an average recognition accuracy rate of 95.08% (standard deviation of 6.12%). The performance in each frequency band is also very good. Compared with the low-frequency band (Delta band, Theta band and Alpha band) features, the high-frequency band (Beta band and Gamma band) features are more related to human brain activity. Compared with DGCNN and CGB-net, the accuracy rate of the whole frequency band is improved by 4.68 and 2.78%, respectively, and the stability of our proposed model is better.

#### Performance on SEED-IV

On the SEED-IV data set, in order to better compare other methods, we have the same settings as Zheng et al. (2018) and Li et al. (2020). Each subject has a total of 24 trials in an experiment. The first 16 trials are selected as the training set, and the remaining 8 trials are used as the test set. The 8 trials in the test set include 2 trials of happy, neutral, sad, and fear.

In order to evaluate the performance of the MDGCN-SRCNN model proposed in this paper on the SEED-IV dataset, we compared the baseline methods SVM, DBN, DGCNN, etc., and also compared the current latest methods RGNN, BiHDM, SST-EmotionNet, etc. We conduct experiments and comparisons on the DE features of the whole frequency band (Delta, Theta, Alpha, Beta, and Gamma). The results are shown in Table 3.

**Table 3:** The accuracy of the proposed method is compared with the existing methods on the SEED-IV dataset.

**Model**	**ACC (%)**	**STD (%)**
SVM (Zhong et al., 2020)	56.61	20.05
DBN (Zhong et al., 2020)	66.77	7.38
GSCCA (Zheng, 2017)	69.08	16.66
DGCNN(Zhong et al., 2020)	69.88	16.29
BiDANN (Li Y. et al., 2018)	70.29	12.63
EmotionMeter (Zheng et al., 2018)	70.58	17.01
BiHDM (Li et al., 2020)	74.35	14.09
RGNN (Zhong et al., 2020)	79.37	10.54
SST-EmotionNet (Jia et al., 2020)	84.92	**6.66**
MDGCN-SRCNN	**85.52**	11.58

*Bold represents the best result*.

In Table 3, it can be seen that the MDGCN-SRCNN model proposed in this paper achieves the most advanced performance at present, with an average accuracy of 85.2%, which is 15.64 and 6.15% higher than the similar graph networks DGCNN and RGNN, respectively. It shows that MDGCN-SRCNN model has a good advantage in emotion recognition task.

### Visualization of Results

**Figure 3:**
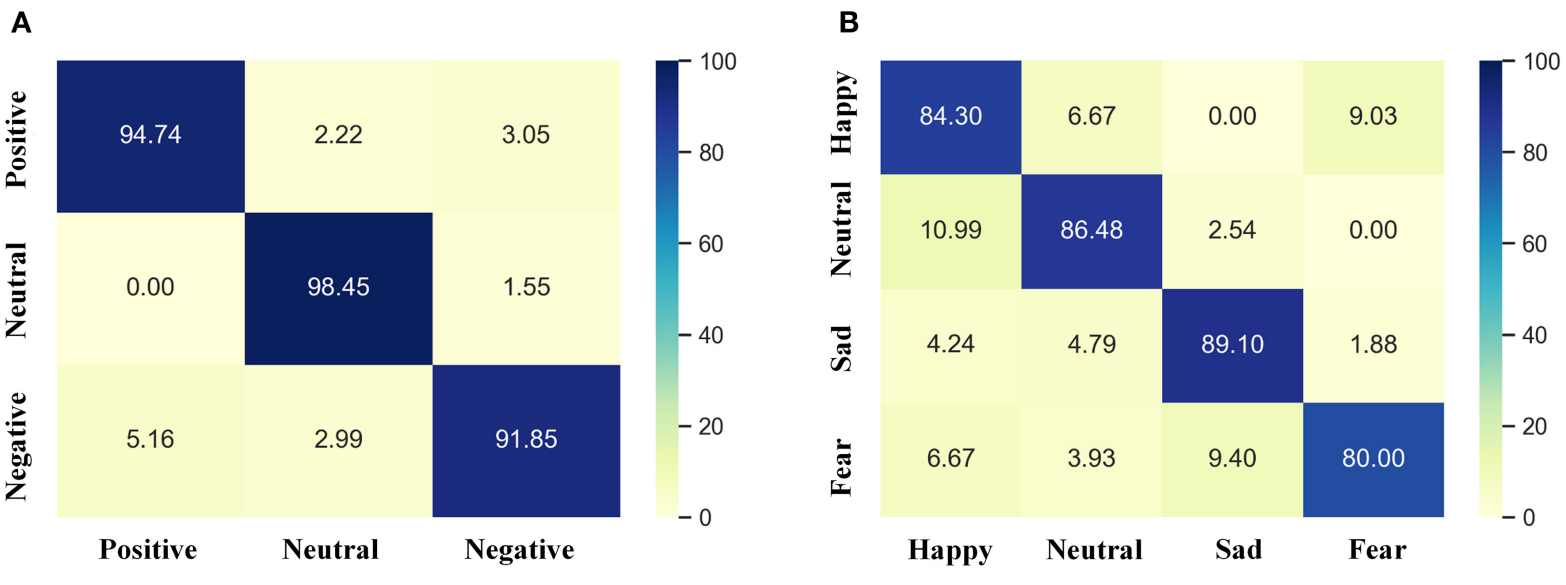
Confusion matrix of different data sets. **(A)** is the confusion matrix of the SEED data set; **(B)** is the confusion matrix of the SEED-IV data set.

In order to intuitively distinguish between different emotions, we draw the confusion matrix of SEED data set and SEED-IV data set. As shown in Figure 3, the positive and neutral emotions of SEED dataset are better distinguished than negative emotions, and the neutral emotions will have certain negative emotions. Fear emotions in the SEED-IV data set are relatively difficult to distinguish. On the contrary, sad emotions are the best to distinguish among the four types of emotions, followed by neutral and happy emotions.

**Figure 4:**
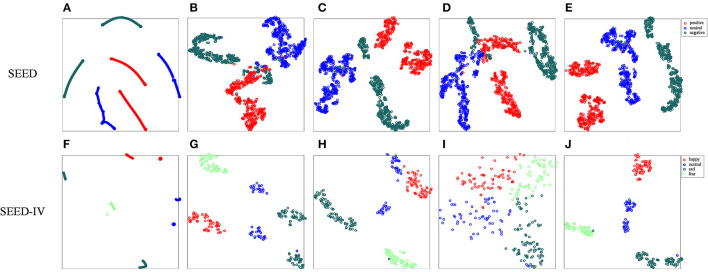
Visualization of t-SNE output from different layers. **(A,F)** are the original data; **(B,G)** are the feature distributions output by the first layer of GCN; **(C,H)** are the feature distributions output by the second layer of GCN; **(D,I)** is the feature distribution of the output of the convolutional neural network; **(E,J)** are the feature distributions after connecting the two layers of GCN and SRCNN. Different colors represent different emotions.

In addition, we performed a visual analysis of feature distribution to evaluate the influence of the corresponding modules in the MDGCN-SRCNN model. We use t-SNE to reduce the dimensionality of the features output in different layers, and draw a two-dimensional feature distribution map. Figure 4 shows the original artificial feature distribution of the SEED data set and the SEED-IV data set and the output feature distribution of different layers. It can be seen from Figure 4 that the output features of a single layer will be confused with some samples to varying degrees, resulting in a decrease in classification accuracy. In addition, the features learned by two-layer GCN are more representative than those learned by single-layer GCN. Moreover, the deep features learned by SRCNN can better express each type of emotion. Therefore, by combining the shallow GCN features and the deep SRCNN features, the features that express various emotions can be fully learned, and the robustness of the model is improved.

### Study of Brain Connection

**Figure 5:**
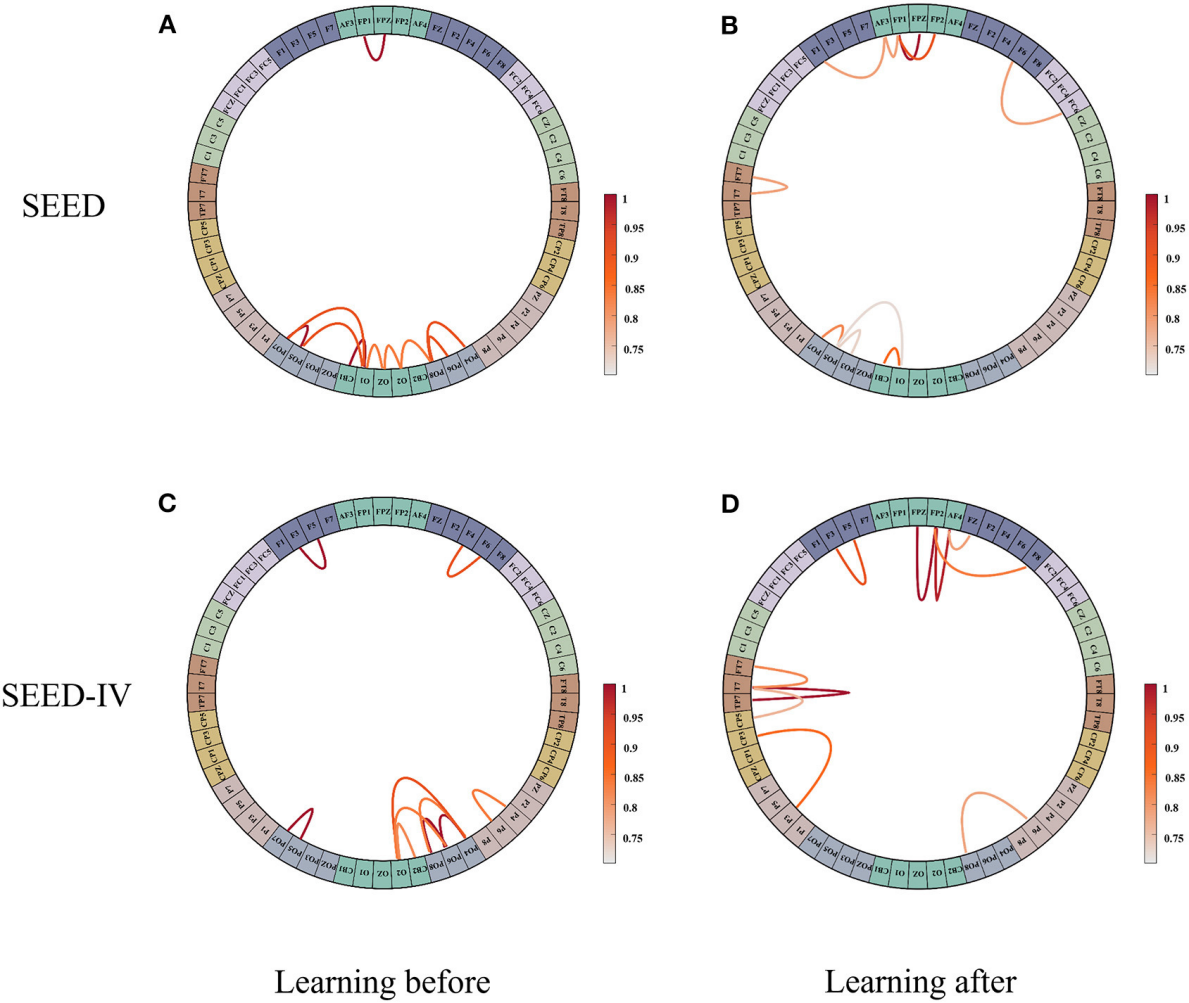
The connection weights between the first 10 channels are selected from the initial adjacency matrix and the learned adjacency matrix. **(A)** is the initial adjacency matrix of the SEED data set, **(B)** is the adjacency matrix learned from SEED dataset. **(C)** is the initial adjacency matrix of the SEED-IV data set, **(D)** is the adjacency matrix learned from SEED-IV dataset.

We analyzed the connections between the brain regions in human emotion. We standardize the initial adjacency matrix and the adjacency matrix learned by network, and the range of their values is [0, 1]. We select the top 10 strongest connection weights in the SEED dataset and the SEED-IV dataset, respectively, and draw their connection diagram, as shown in Figure 5. Figures 5A,B show the initial connection and the learned connection selected on the SEED data set, respectively. Figures 5C,D show the initial connection and the learned connection selected on the SEED -IV data set, respectively. It can be seen from Figures 5A,C that the initial connection between the left and right hemispheres of the brain is symmetrical and concentrated in the occipital lobe, while the subjects' movie clips are mainly visual stimulation, and the visual information is mainly processed in the occipital lobe, which is in line with the common sense. After learning, the connection between the left and right hemispheres of the brain becomes asymmetric, as shown in Figures 5B,C, especially in the temporal lobe, frontal lobe, and parietal lobe, where the asymmetry is the strongest, indicating that these regions are crucial to emotional activity. Among the local connections, (FT7-T7), (FP2-FPZ), (FP2-AF4), and (T7-TP7) are the strongest connections, and in the global connection (FP1-FP2), is the strongest connection. It shows that emotional activities in the brain are mainly local connections, and global connections are complementary connections. In addition, the more complex emotions are, the more brain areas need to be used. The more complex the connections between brain areas, the greater the strength of local connections.

In order to explore the impact of the initial method of the adjacency matrix**A**on the performance of the model, we chose the common initial methods, such as phase locking (PLV), Pearson correlation coefficient (PCC), local, and global connections used in RGNN and in [0,1] and random values. We extracted DE features in the SEED data set and SEED-IV data set for comparison. Table 4 shows the effect of using different initial methods of adjacency matrix on the performance of the MDGCN-SRCNN model on the SEED dataset and the SEED-IV dataset. The results show that using PCC as the initialization method of the adjacency matrix achieves the best performance. In RGNN, a global connection is added on the basis of relative physical distance, and a great improvement has been made on the SEED-IV data set. The performance of PLV as an initialization method of the adjacency matrix is equivalent to that of random value selection.

**Table 4:** The SEED data set and SEED-IV data set are compared by using different adjacency matrix A initialization methods.

**Method**	**SEED**		**SEED-IV**	
	**ACC(%)**	**STD(%)**	**ACC(%)**	**STD(%)**
PCC	**95.08**	**6.12**	**85.52**	11.58
RGNN	91.98	7.21	84.16	**10.93**
PLV	92.04	7.56	80.92	13.48
Random	91.83	8.47	82.39	11.74

*Bold represents the best result*.

### Ablation Results

In order to verify the contribution of each module of our proposed model, we conducted a series of ablation experiments. The results are shown in Table 5. After removing the SRCNN module, the performance is significantly reduced. The accuracy on SEED and SEED-IV decreased by 3.7 and 4.37%, respectively, indicating the importance of CNN in extracting deep abstract features related to emotion. In addition, the accuracy on SEED and SEED-IV decreased by 1.72 and 1.89% respectively after removing the SRM module, which proved that the attention mechanism such as SRM module can effectively emphasize emotion related features and abandon useless features, so as to improve the recognition performance of the model. Compared with the one-layer GCN, the recognition performance of two-layer GCN on SEED and SEED-IV is improved by 1.66 and1.42%, respectively, indicating that there is a certain complementarity between global features and local features.

**Table 5:** The results of ablation experiments on SEED and SEED-IV (mean/std), “~” represents the module is removed.

**Model**	**SEED**	**SEED-IV**
MDGCN-SRCNN	**95.08/**6.12	**85.52/**11.58
~SRM	93.36/6.49	83.63/10.20
~SRCNN	91.38/7.74	81.15/10.89
One-layer GCN	89.72/6.52	79.73/9.61

*Bold represents the best result*.

## Conclusions

In this paper, we propose a multi-layer dynamic graph convolutional network-style-based recalibration convolutional neural network (MDGCN-SRCNN) model for EEG-based emotion recognition. In our model, EEG data is considered to be non-Euclidean structure, and dynamic graph neural network is used to learn the connection relationship between each channel of EEG signal as a shallow layer feature. Because analyzing emotions through EEG signals is very complicated. We use a style-based recalibration convolutional neural network to further extract abstract deep layer features. Finally, the fully connected layer is used to search for the features most relevant to emotions in the shallow layer and deep layer features for recognition. We conducted systematic experimental verification on the SEED data set and the SEED-IV data set. MDGCN-SRCNN model has achieved better performance on the two public data sets, surpassing the state-of-the-art RGNN. The recognition accuracy on the SEED data set and SEED-IV data set is 95.08 and 85.52%, respectively, and the standard deviation is 6.12 and 11.58%, respectively. Based on using PCC as the initialization method of the adjacency matrix, the MDGCN-SRCNN model is used to learn the local connections and global connections that are most relevant to emotions, such as (FT7-T7), (FP2-FPZ), (FP2-AF4), (T7-TP7), and (FP1-FP2), these connections are mainly distributed in the temporal lobe, frontal lobe, and parietal lobe, proving that these brain regions play a vital role in inducing emotions. In addition, we also found that the more complex emotions are processed, the more brain regions are involved, the more complex the connections, and the greater the strength of local connections.

It is worth noting that using different initial methods of adjacency matrix has a great influence on the connection relationship between graph neural network learning and task. Therefore, it is very important to build the initial connection relationship related to the task. In the future, our main work direction is to build more complex network based on GCN to solve the differences between subjects. And further explore the differences of adjacency matrix under different emotional states, and then analyze the differences of brain activity under different emotional states.

## Data Availability Statement

The original contributions presented in the study are included in the article/supplementary material, further inquiries can be directed to the corresponding author/s.

## Ethics Statement

Written informed consent was obtained from the individual(s) for the publication of any potentially identifiable images or data included in this article.

## Author Contributions

GB was mainly responsible for research design, data analysis, and manuscript writing of this study. KY was mainly responsible for data analysis. LT and BY was mainly responsible for research design. JS was mainly responsible for data collection and production of charts. RZ was mainly responsible for production of charts. LW was mainly responsible for data analysis and document retrieval. YZ was mainly responsible for data collection and manuscript modification. All authors contributed to the article and approved the submitted version.

## Funding

This work was supported in part by the National Key Research and Development Plan of China under Grant 2017YFB1002502, in part by the National Natural Science Foundation of China under Grant 61701089, and in part by the Natural Science Foundation of Henan Province of China under Grant 162300410333.

## Conflict of Interest

The authors declare that the research was conducted in the absence of any commercial or financial relationships that could be construed as a potential conflict of interest.

## Publisher's Note

All claims expressed in this article are solely those of the authors and do not necessarily represent those of their affiliated organizations, or those of the publisher, the editors and the reviewers. Any product that may be evaluated in this article, or claim that may be made by its manufacturer, is not guaranteed or endorsed by the publisher.
